# Micro-CT and machine learning: a high-throughput alternative to histology for follicle reserve assessment in cryopreserved ovarian tissue

**DOI:** 10.1186/s13048-025-01897-8

**Published:** 2025-12-23

**Authors:** Katri Knuus, Mai Nguyen, Markus Hannula, Jasmin Hassan, Marjut Otala, Timo Tuuri, Karolina Lundin, Atte Lahtinen, Pauliina Damdimopoulou, Jari Hyttinen, Kirsi Jahnukainen

**Affiliations:** 1 Faculty of Medicine, University of Helsinki, Helsinki, Finland; 2 Department of Obstetrics and Gynecology, Helsinki University Hospital, Helsinki, Finland; 3 Computational Biophysics and Imaging Group, Faculty of Medicine and Health Technology, Tampere University, Tampere, Finland; 4 Department of Women’s and Children’s Health, Karolinska Institutet, Stockholm, Sweden; 5 Department of Gynecology and Reproductive Medicine, Karolinska University Hospital, Stockholm, Sweden; 6 Applied Tumor Genomics Research Program, Faculty of Medicine, University of Helsinki, Helsinki, Finland; 7 Department of Medical and Clinical Genetics, Medicum, Faculty of Medicine, University of Helsinki, Helsinki, Finland; 8 Children’s Hospital and Pediatric Research Center, Helsinki University Hospital, Helsinki, Finland

**Keywords:** Ovarian tissue cryopreservation, Ovary, Micro-CT, Follicular density, 3D analysis

## Abstract

**Background:**

Ovarian tissue cryopreservation followed by transplantation after cancer remission is a fertility preservation strategy available for certain patient groups, such as pre-pubertal and adolescent girls, as well as adult females requiring urgent gonadotoxic therapy. Quantitative assessment of follicular density in cryopreserved cortical tissue is critical for evaluating tissue quality and estimating its reproductive potential. Conventional analysis, based on manual follicle counts in serial histological sections, is time-consuming, labor-intensive, and prone to variability from uneven follicle distribution and inconsistent tissue orientation. To address these limitations, we developed a high-throughput, automated method combining micro-CT, machine learning, and morphological analysis to quantify oocyte density and other morphological features throughout entire ovarian cortical tissue samples.

**Results:**

Three-dimensional segmentation analysis enabled quantification of oocyte density in the samples within the cortical region 1 mm below the surface epithelium. Oocytes in pediatric samples were located significantly closer to the surface compared to those in adult tissue, with median distances of 139.4 μm and 370.2 μm, respectively (*P* < 0.0001) and exhibited markedly higher local oocyte neighbor counts, with median values of 6 and 2 in pediatric and adult tissues, respectively (*P* < 0.0001), consistent with higher oocyte density and clustered spatial organization in younger individuals. Simulated histology using every 10th virtual sections —corresponding to 40 μm separated histology slices— closely approximated full-volume micro-CT estimates of oocyte density. Analysis based on only five virtual sections aligned with micro-CT data exclusively in pediatric samples with high oocyte density, whereas in adult samples it led to substantial inaccuracies in oocyte density estimation.

**Conclusion:**

Micro-CT scanning combined with machine learning analysis represents a novel high-throughput and automated approach for estimating oocyte count in cryopreserved ovarian cortical samples. In addition, three-dimensional analysis offers valuable insights into oocyte localization and spatial distribution within the ovarian cortex, presenting a promising alternative to conventional histology for future clinical and research applications.

**Supplementary Information:**

The online version contains supplementary material available at 10.1186/s13048-025-01897-8.

## Introduction

Ovarian tissue cryopreservation (OTC) represents an established fertility-preserving strategy for patients undergoing gonadotoxic therapies [1], used especially for young girls, adolescents and patients requiring urgent medical treatment [2–4]. Cryopreserved ovarian cortical tissue retains endocrine functionality and, upon thawing and autologous transplantation, can restore ovarian function in otherwise menopausal and infertile individuals [5]. The tissue can be stored long-term —potentially for decades— until the patient is ready for reimplantation. Quantification of follicular density in cryopreserved cortical samples is critical for evaluating tissue quality and predicting future fertility potential. The number of primordial follicles in ovarian samples varies by age, diagnosis, and prior treatments. Reference values for follicle densities in different age groups, along with a Z score calculator to standardize the histological evaluation of the oocyte reserve in cortical tissue pieces have recently been published [6, 7]. In this model, nongrowing follicles are counted within 1 mm of the ovarian surface, which is the area recommended by recent guidelines, in order to standardize mean follicle density (MFD) calculations [8]. However, cortical thickness in prepubertal ovaries remains undefined, which may limit the precision and wider applicability of the reference model and MFD calculations in pediatric populations.

Follicles are unevenly distributed within the human ovarian cortex [9–11], with heterogeneity increasing with age [12]. According to recommendations, MFD should be calculated from the entire ovarian cortex to ensure precise evaluation. However, in clinical fertility preservation this is not feasible, as the majority of the tissue is preserved for the patient and MFD estimation relies only on a small sample [8]. Currently, the standard approach for assessing follicle density relies on histological sectioning and manual counting of a few sections. To improve the precision of follicle density estimation, a mathematical algorithm incorporating a correction factor has been developed and validated [6, 7]. Originally proposed by Schmidt et al. [11], the correction factor reduces the potential double-counting of follicles in serial histological sections and enables reliable calculations across varying sectioning intervals. Despite its utility, histological morphometric analysis remains time-consuming, laborious, and prone to inter-observer variability. Additionally, it requires the use of CMR (Carsinogenic, Mutagenic and Reprotoxic) classified fixatives. Recent advances have focused on automating follicle quantification from histological slides using artificial intelligence [13–16], thereby enhancing both efficiency and reproducibility. Nevertheless, these automated approaches still depend on serial histological sections. The reliability of current histological methods for clinical fertility preservation is further limited by small sample sizes and inconsistent tissue orientation during embedding, which can hinder accurate identification of the cortical region. Alternative approaches for estimating follicle amount include enzymatic digestion with calcein staining, followed by assessment with a fluorescent microscope [17]; staining with rhodamine 123, followed by laser scanning confocal microscopy; and stereomicroscopic imaging on glass-bottom dishes [18]. However, these methods are labor-intensive, allow limited volumetric quantification, and often involve cytotoxic reagents, making them impractical for clinical application.

Micro-computed tomography (micro-CT) enables high-resolution, three-dimensional imaging of biological tissues at micrometer-scale resolution, shown e.g. in intestine or cancer biopsy imaging [19, 20]. It also offers the advantage of post-acquisition reorientation and 3D morphometric analysis of the scanned tissue. Several studies have employed micro-CT for imaging ovarian biopsies, both in various animal models [21–23] and in humans [24]. These investigations have generally succeeded in identifying key anatomical structures such as the cortex, medulla, and antral follicles. Notably, some studies [22, 24] have also reported the visualization of early-stage follicles. However, none of these studies have provided a comprehensive assessment of the cortical oocyte reserve. This requires high imaging resolution and advanced segmentation techniques to accurately identify and quantify primordial and primary follicles in the ovarian cortex.

**Fig. 1:**
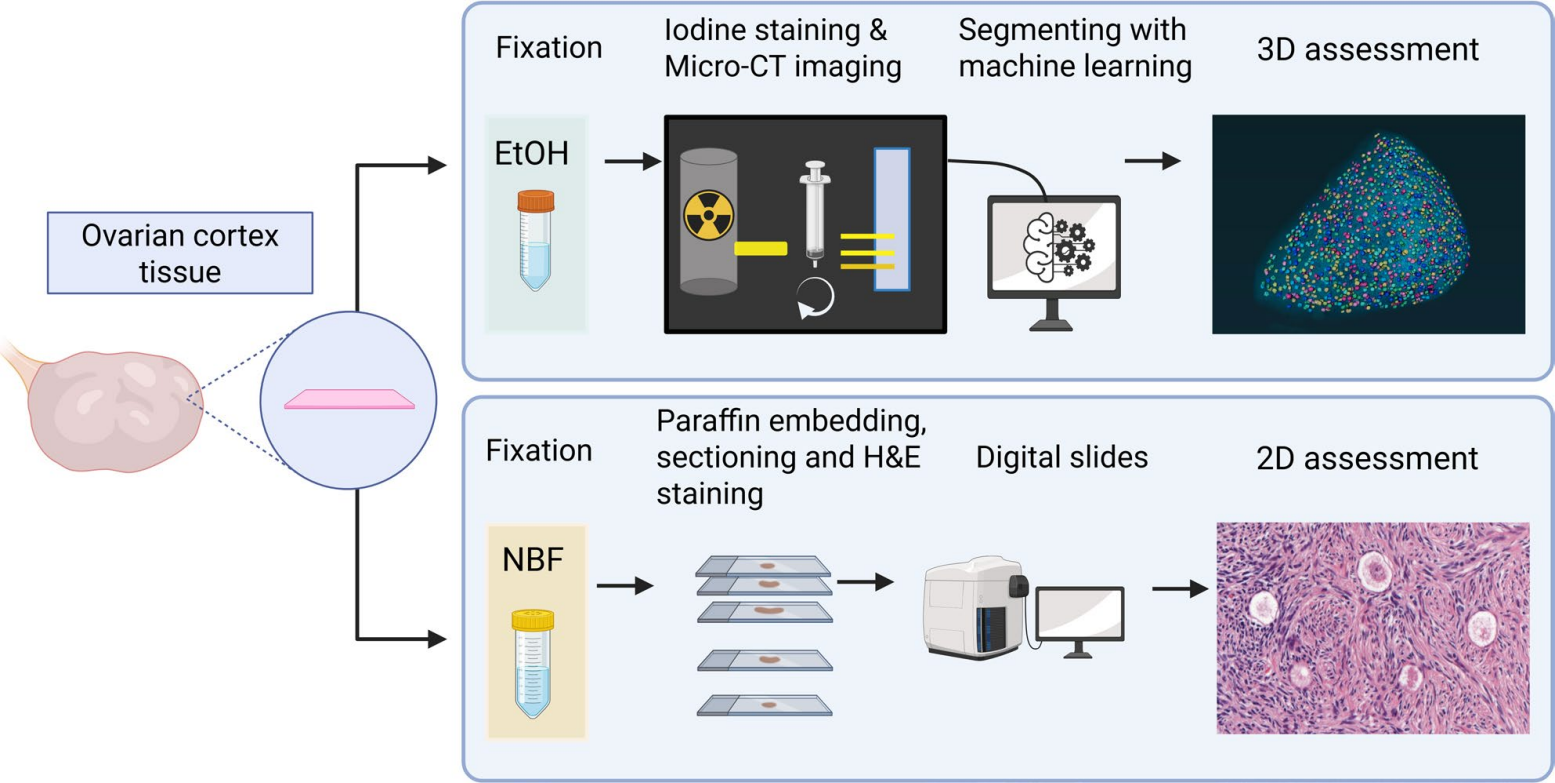
Experimental setup. Ovarian cortical tissue was obtained from both pediatric and adult patients. Two representative tissue fragments were processed separately: one fixed in 10% neutral-buffered formalin and the other in 70% ethanol. The formalin-fixed samples were paraffin-embedded, serially sectioned, and every 10th section was stained with hematoxylin and eosin. Digital scans of the stained sections were used to quantify oocyte density via conventional histological assessment. The ethanol-fixed samples were stained with buffered Lugol’s solution, imaged using high-resolution micro-computed tomography (micro-CT), and subjected to automated segmentation using a machine learning–based algorithm and morphometric analysis. Oocyte density and other geometry features was then quantified through volumetric three-dimensional analysis of the reconstructed cortical tissue. Created in BioRender. Knuus, K. (2025) https://BioRender.com/ovsz93t

The objective of the present study was to develop a novel high-throughput, automated approach for quantifying oocyte density in ovarian cortical strips by integrating micro-CT imaging with machine learning-based analysis. We present a method for three-dimensional quantification of oocyte density, localization, and spatial distribution in both pediatric and adult ovarian cortical tissue (Fig. 1). The performance of the micro-CT method was compared with conventional histology and simulated histology based on virtual sections derived from full-volume micro-CT data.

## Materials and methods

### Patient samples

Adult ovarian samples were obtained during gender reassignment surgeries.

Ovarian samples from children and adolescents were collected as part of fertility preservation programs. OTC was offered to patients at high or very high risk of premature ovarian insufficiency due to planned treatments, such as allogeneic/autologous hematopoietic stem cell transplantation or radiotherapy involving the ovaries. This was done in accordance with fertility preservation guidelines from the Nordic Society of Paediatric Haematology and Oncology (NOPHO, 2013) and Swedish national recommendations for infertility risk assessment. As OTC is still considered experimental in pediatric patients, one-third of the ovarian tissue was donated for research purposes.

Ovarian tissue was collected from five pediatric patients (ages 2–16) and six adult patients (ages 23–39) at either Helsinki University Hospital or Karolinska University Hospital in Stockholm. All pediatric patients were diagnosed with cancer or severe hematological disorders. Basic patient characteristics, including age, diagnosis, and cancer treatment prior to OTC, are summarized in Table 1. Exposure to alkylating agents was calculated using the mean cumulative cyclophosphamide equivalent dose (CED) [25], while exposure to anthracyclines was assessed using the doxorubicin isotoxic equivalent (DIE) with the following conversion factors: 1 for doxorubicin, 0.5 for daunorubicin, 0.67 for epirubicin, 5.0 for idarubicin, and 10 for mitoxantrone [26]. All adult patients had received androgen therapy prior to surgery.

**Table 1:** Clinical characteristics, patient group and diagnosis of the patients (*n* = 11)

Sample ID	Patient group	Age years	Diagnosis	previous exposure to cancer therapy	CED	DIE
C1	fertility preservation	2	CNS tumor	yes	7992	78
C2	fertility preservation	4	CNS tumor	yes	0	0
C3	fertility preservation	10	Benign hematological disease	no	0	0
C4	fertility preservation	12	Leukemia	yes	8176	320
C5	fertility preservation	16	Non-CNS tumor	no	0	0
A1	ovariectomy	23	transgender			
A2	ovariectomy	31	transgender			
A3	ovariectomy	31	transgender			
A4	ovariectomy	32	transgender			
A5	ovariectomy	34	transgender			
A6	ovariectomy	39	transgender			

### Tissue processing and histological analysis

In children, a maximum of one-third of the total ovarian volume was removed and stored in Dulbecco’s phosphate-buffered saline (DPBS without calcium and magnesium; Gibco, Thermo Fisher Scientific, Paisley, UK) on ice for transport. In adult patients, both ovaries were removed during surgery, and approximately half of each ovary was transferred on ice in DPBS to the research facility. The soft medullary tissue was manually trimmed using a scalpel, and the remaining cortical tissue was cut into smaller pieces. A small cortical fragment from each ovarian sample was fixed in formalin and embedded in paraffin. The samples were then sectioned into 4 μm thick serial sections, with every 10th section stained with hematoxylin and eosin and digitized using either a Pannoramic scanner (3DHistech) or a Zeiss Axioscan 7.

Digitized sections were analyzed using Pannoramic Viewer (version 1.15.4, 3DHistech) or QuPath [27] as previously reported [6, 7]. Unilaminar follicles were counted within 1 mm of the surface epithelium. Between three and fifteen sections per sample were evaluated. A subset of samples was independently assessed by two observers, with interobserver concordance exceeding 97%. The total number of oocytes and the total cortical volume were extrapolated from the analyzed sections, and oocyte density was calculated using these values, applying a modified version of the correction formula originally proposed by Schmidt et al. [11], as detailed in the earlier publication [6, 7].

### Micro-CT imaging

A small cortical piece of each ovarian sample was fixed in 70% ethanol and subsequently immersed in buffered iodine solution (B-Lugol), prepared according to the method described by Dawood et al. [28], for three days. The sample was then placed inside a 1 ml syringe containing B-Lugol and secured between two pistons to stabilize it and prevent movement. The samples were imaged with X-ray micro-computed tomography to reveal their three-dimensional (3D) structure. A MicroXCT-400 device (Carl Zeiss X-ray Microscopy Inc., Pleasanton, CA, USA) was used with a 40 kV tube voltage and 250 µA current. 1601 projections were taken around the sample with a 10-second exposure time. Projections were reconstructed into a 3D volume using XMReconstructor software, resulting in a 2.28 μm voxel size. The staining process required a total of three days, followed by approximately four hours of imaging. However, the actual hands-on time per sample was limited to about 30 min.

### Machine learning analysis from each sample

The reconstructed image data was presented as a 3D image, allowing virtual slicing along any axis. For machine learning analysis, U-Net —a convolutional neural network designed for biomedical image segmentation— was used due to its ability to achieve high accuracy with relatively small training datasets [29]. The model was initially pretrained on a related CT scan dataset [30] and subsequently fine-tuned using ovarian micro-CT images acquired in this study. For fine-tuning, 10 to 15 slices from each of the 16 scanned samples were randomly selected along a single axis and manually segmented by expert using ITK-SNAP [31]. These annotated slices were divided into training and validation sets (Supplementary Fig. 1), while the remaining slices from each sample were reserved for testing. Given the limited dataset, data augmentation was applied to the training and validation sets. Augmentation techniques included the addition of noise, image rotations by 45 and 90 degrees, and mirror transformations. After the testing phase, five samples were excluded due to non-uniform quality across their full volume. Once trained, the model was applied to segment oocytes from the entire volume of the test set samples. All stages of the model pipeline were implemented in Python using the TensorFlow framework. Training was conducted with a learning rate of 0.0001 for 25 epochs and a batch size of 16. The quantitative validation of the model was done with DICE, precision and recall (Supplementary Table 1).

Post-processing was conducted in Avizo 2023.1 (Thermo Fisher Scientific, Waltham, MA, USA). Segmentation accuracy was manually reviewed slice by slice using the Color Wash function. Post-processing included removal of blood vessels, separation of merged oocytes, and elimination of noise artifacts. Quantitative analysis was performed using Avizo’s toolbox to extract total oocyte counts and cortical volume, with the cortex defined as the region within 1 mm of the surface epithelium. The distance of each oocyte from the surface epithelium was measured, except in sample A5 where the epithelium was not clearly visible. Additionally, the number of neighboring oocytes within a 40 μm radius was calculated for each oocyte —a threshold previously reported as the maximum distance for follicular neighbor effects [32].

Visualization of a sample in the video was done with Dragonfly software version 2024.1 (Comet Technologies Canada Inc., Montreal, Canada).

### Simulation of histology using virtual sections

The current gold standard for oocyte quantification is based on sparsely sampled histological sections. To evaluate the accuracy of this method, high-resolution micro-CT datasets were used to generate virtual cross-sections as digital surrogates for conventional histology slides. From these, oocyte counts, diameters of the largest 10% of oocytes, and section areas were extracted and applied to a previously validated mathematical model for follicle density estimation [6, 7], which includes a correction factor [11], to account for duplicate follicle counts in serial sections. To replicate standard histological sampling, section thickness was set at 4 μm, with every 10th section analyzed. Two sampling strategies were applied: comprehensive sampling across the entire sample volume or a limited sampling of five virtual sections (Sects. 10, 20, 30, 40, and 50). The cortex was defined as the region within 1 mm of the surface epithelium.

Virtual sectioning enabled systematic analysis of factors affecting histological quantification. Variability in oocyte density estimates was assessed by varying the starting point of five-section series, and the robustness of the density estimation algorithm [6, 7] was tested across different sectioning intervals. All analysis were conducted using Matlab E2022b (The MathWorks Inc., Natick, MA, USA).

### Statistical analysis

Statistical analysis was performed using GraphPad Prism (version 10.1.2 (324)). The Mann–Whitney test was used to compare the child and adult groups. All significance tests were two-tailed, and p-values < 0.05 were considered statistically significant.

## Results

### Quantification of oocytes in 3D and comparison with light microscopy-based histology

**Fig. 2:**
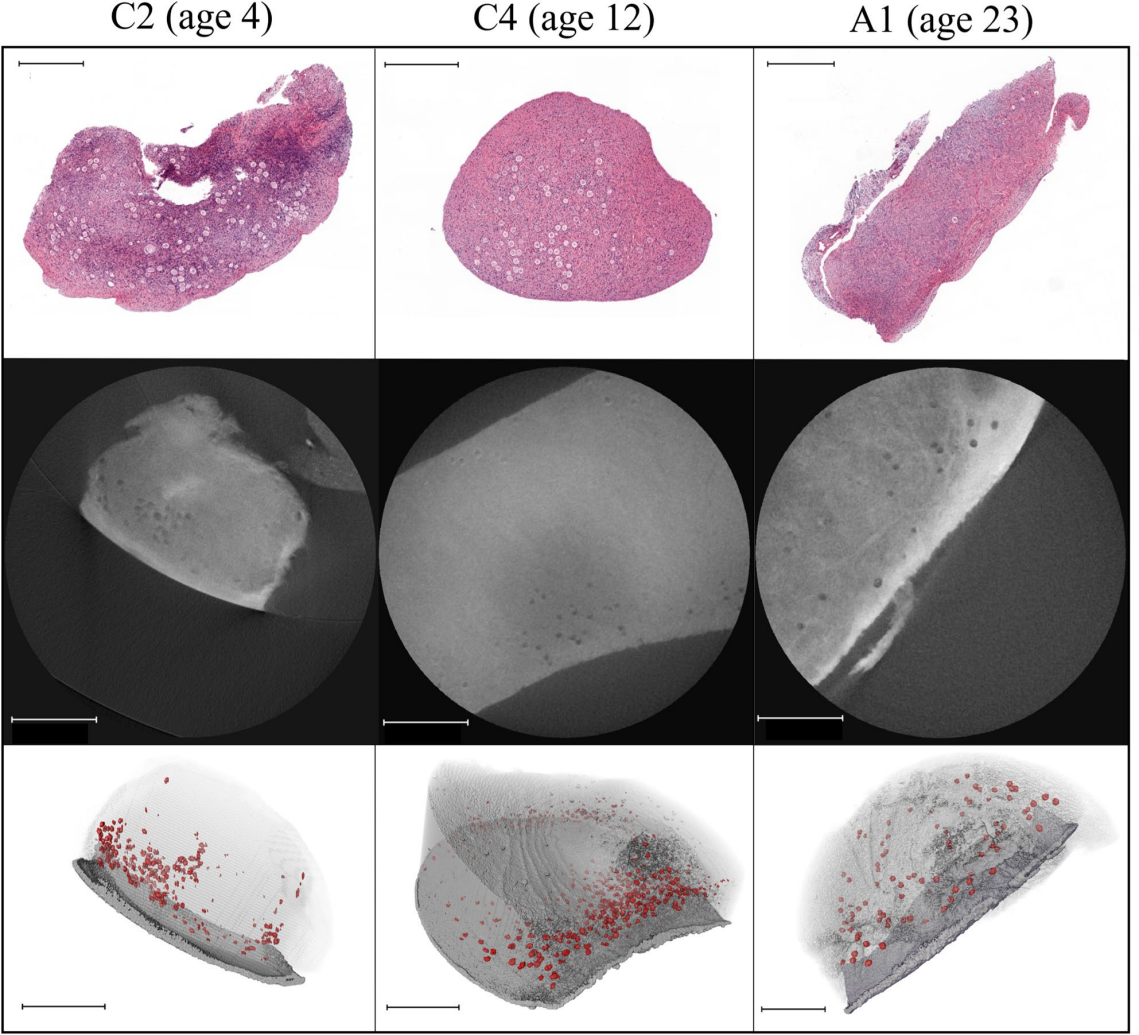
Representative histological sections, micro-CT imaging, and three-dimensional reconstructions of ovarian cortical tissue from patients aged 4 (C2), 12 (C4) and 23 (A1). The upper panel shows hematoxylin and eosin-stained formalin-fixed histological sections. The middle panel depicts example of virtually sectioned 2D micro-CT images, in which oocytes are identifiable as discrete hypodense (dark) spherical structures, and the surface epithelium as a hyperdense (bright) peripheral layer in samples C2 and A1. The lower panel illustrates three-dimensional reconstruction of the corresponding tissue samples, with the surface epithelium rendered in opaque gray, cortical tissue in semi-transparent gray, and the detected oocytes visualized in red. All scale bars in the images are 500 μm

Micro-CT combined with machine learning–based segmentation was employed to generate high-resolution three-dimensional reconstructions of cortical ovarian tissue samples, enabling the identification and spatial localization of oocytes within primordial follicles (Fig.2; Additional Video 1.)

**Fig. 3:**
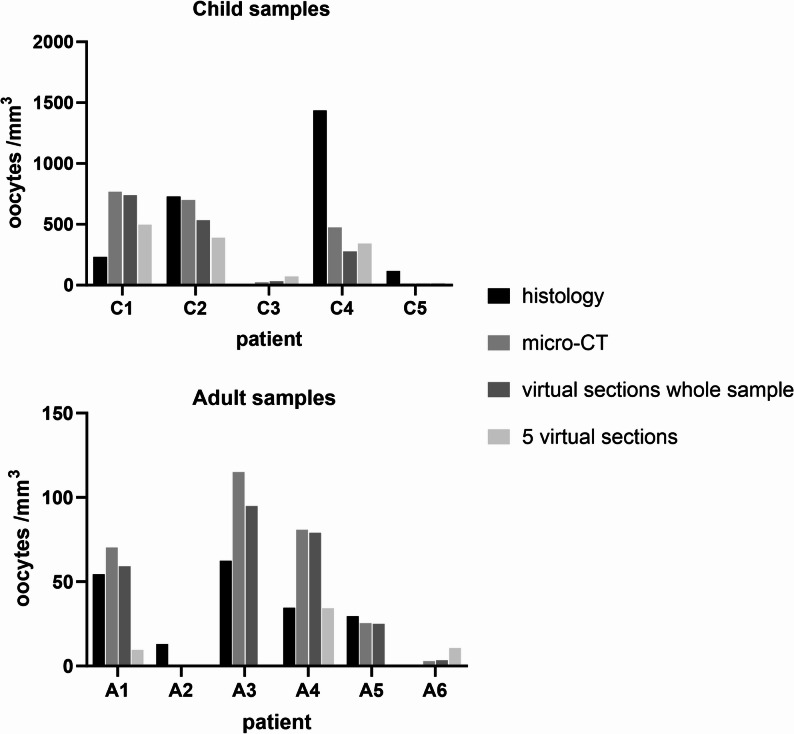
Comparative analysis of oocyte density as determined by conventional histology, micro-computed tomography (micro-CT), and simulated histological assessment based on virtual micro-CT sections. The upper panel presents data from pediatric ovarian cortical samples, and the lower panel from adult samples. Oocyte densities from conventional histology (black bars) were derived from hematoxylin and eosin–stained serial sections of formalin-fixed tissue. Densities from 3D analysis and virtual sections (gray bars) were obtained from a separate ethanol-fixed tissue fragment from the same individual, imaged with micro-CT. Virtual simulated histology was performed on the micro-CT dataset using either every 10th virtual section across the entire sample or a subset of the first five virtual sections to approximate conventional histological sampling

Segmentation analysis enabled quantification of the total oocyte count and the volume of the ovarian cortical region, defined as the zone extending 1 mm beneath the surface epithelium. Cortical oocyte density was subsequently calculated for each sample and compared to corresponding values obtained via conventional light microscopy-based histological assessment (Fig.3). However, direct correlation between micro-CT–derived and histology-derived follicle densities was not feasible, as the tissue samples used for histological evaluation and micro-CT imaging were not identical. Substantial inter-sample variability in cortical density within the tissue samples from the same patient was observed. In the micro-CT–based analysis, oocyte density was derived from the entire cortical biopsy volume, providing a comprehensive volumetric assessment compared to histology. Importantly, micro-CT was able to detect oocytes also in patients with very low absolute follicle count. Our findings demonstrate that micro-CT–derived oocyte density measurements are comparable to, or exceed, those obtained through conventional histology in adult ovarian tissue. Furthermore, micro-CT scanning combined with machine learning-based segmentation offers excellent sensitivity for detecting low-frequency oocytes. An exception was sample A2, in which no oocytes were detected in the entire specimen analyzed by micro-CT.

### Oocyte distance to the surface epithelium

**Fig. 4:**
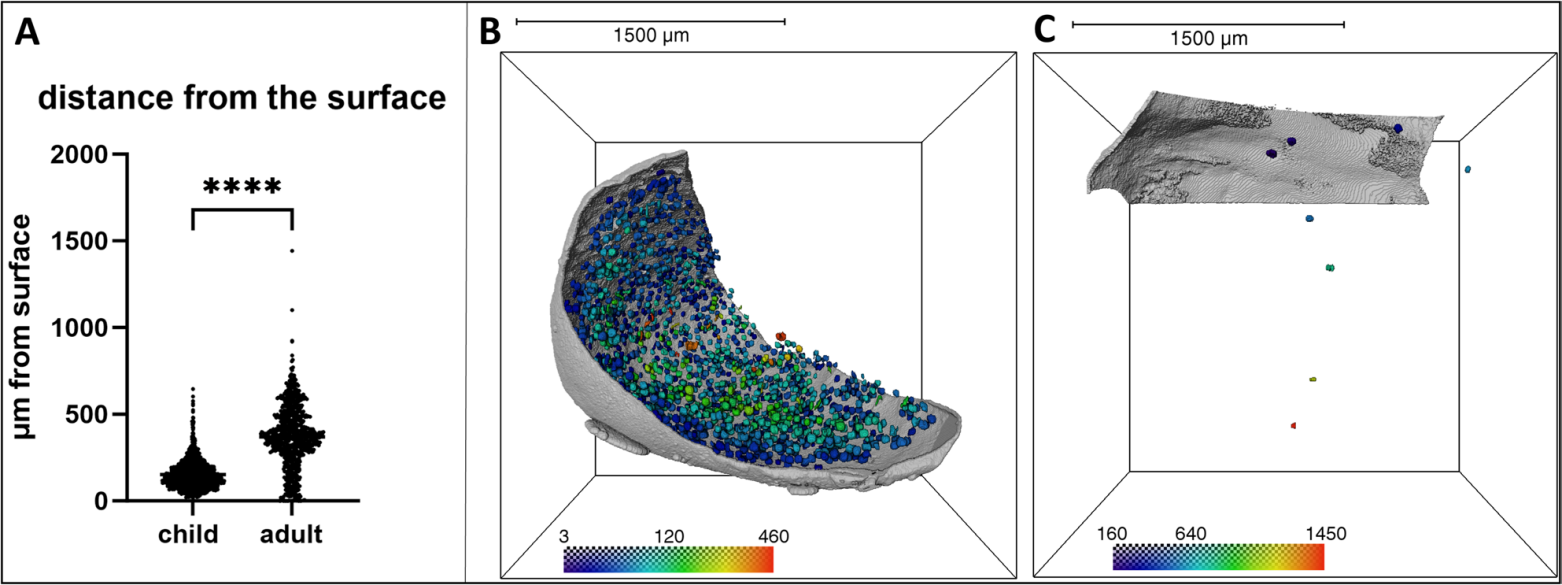
Quantitative analysis of oocyte distance from the ovarian surface epithelium in pediatric and adult cortical tissue samples. **A** The distance from the ovarian surface epithelium was measured for each individual oocyte in five pediatric samples (*n* = 2,465 oocytes) and four adult samples (*n* = 908 oocytes). Oocytes in pediatric tissue were significantly closer to the surface epithelium compared to those in adult tissue, with median distances of 139.4 μm and 370.2 μm, respectively. (*P* < 0.0001, Mann–Whitney U test). **B** Spatial distribution of oocytes in a representative pediatric sample from a 2-year-old patient (C1), with distance from the surface epithelium (rendered in grey) visualized via color mapping. **C** Corresponding visualization in a sample from a 39-year old adult patient (A6). The color scale indicates the radial distance from the surface epithelium in micrometers. In pediatric tissue, oocytes are densely distributed near the surface, whereas in adult tissue, oocyte numbers are markedly reduced and more deeply located within the cortical region

To characterize the spatial distribution of oocytes in relation to the ovarian surface epithelium, the epithelial boundary was segmented from the micro-CT datasets, and the distance from the surface to each individual oocyte was quantitatively measured. Our results show that in pediatric ovarian cortex, oocytes were localized closer to the surface epithelium, with a median depth of 139.4 μm (range: 0.13–645.6 μm), compared to adult cortical tissue, where oocytes exhibited a deeper distribution pattern with a median depth of 370.2 μm (range: 0.04–1443.0 μm) (Fig.4). In both pediatric and adult ovarian cortex samples, most oocytes were located within 1 mm of the surface epithelium, corresponding to the typical thickness of cortical tissue fragments used for cryopreservation in clinical settings (Fig. 4).

### Clustering of the oocytes

**Fig. 5:**
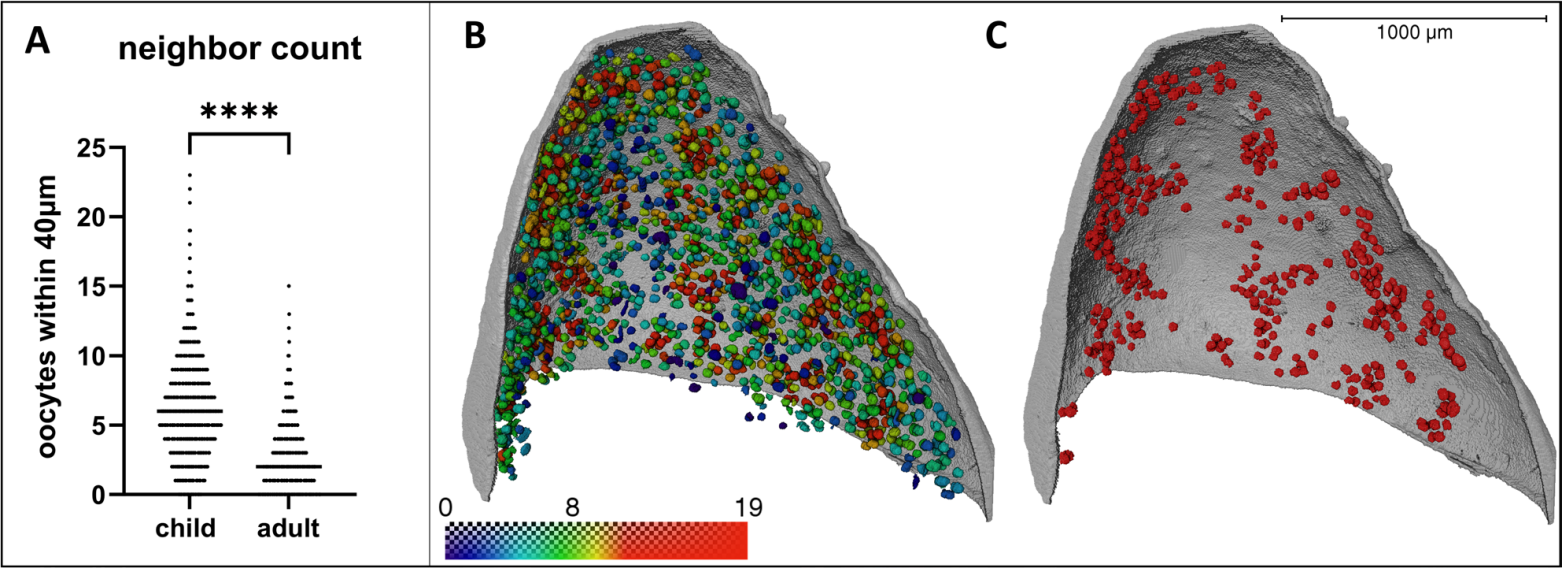
Quantitative analysis of oocyte neighbor counts and spatial clustering in ovarian cortical tissue. **A** Comparison of local oocyte neighbor counts between five pediatric (*n* = 2,465 oocytes) and five adult (*n* = 922 oocytes) ovarian cortex samples. Pediatric samples demonstrated significantly higher neighbor counts compared to those in adult tissue, with median numbers of 6 and 2, respectively. (*P* < 0.0001, Mann–Whitney U test). **B** Spatial mapping of a representative pediatric sample from a 2-year-old patient (C1), with individual oocytes color-coded based on the number of neighboring oocytes located within a 40 μm radius. **C** The same sample visualized to display only oocytes possessing ≥ 10 neighbors, revealing distinct focal clustering patterns characteristic of the pediatric ovarian cortex

To assess the spatial organization and clustering of oocytes within the ovarian cortex, we quantified the number of neighboring oocytes surrounding each individual oocyte. Any oocyte located within a 40 μm radius was counted as neighbor. Pediatric samples exhibited a significantly greater number of neighboring oocytes per oocyte compared to adult samples, consistent with the higher follicular density observed in younger individuals. Furthermore, the spatial distribution of oocytes was non-random and characterized by localized clustering, rather than homogeneous dispersion. This pattern of aggregation was evident even in pediatric samples, suggesting that follicular clustering is an intrinsic feature of cortical organization (Fig. 5).

### Simulated histology using virtual sections from micro-CT data

As the tissue specimens analyzed by micro-CT and histology were not identical, a simulated histological analysis was also conducted using the micro-CT datasets. Based on our results, analysis using every 10th virtual section produced density estimates that closely approximated those obtained from whole-volume micro-CT analysis (Fig. 3). In contrast, analysis based on a limited set of five virtual sections yielded oocyte density estimates consistent with full micro-CT results only in pediatric samples, where follicle distribution was relatively homogeneous and density was consistently high. In adult samples, which contained fewer follicles and exhibited greater spatial heterogeneity, the estimates diverged substantially. To illustrate this variability, we assessed how the oocyte estimates changed when the five-section series was initiated from different locations within the sample. In adult tissues, oocyte density was frequently over- or underestimated, depending on the spatial distribution of follicle clusters (Additional Fig. 2) 

To assess the robustness of the histology-based oocyte density estimation algoritm, uncorrected values from 4 μm virtual sections were compared with algoritm corrected estimates and 3D micro-CT measurements (Additional Fig. 3). The uncorrected values were approximately ten times higher than the corrected and 3D-derived estimates, reflecting overestimation due to duplicate follicle counts. The algorithm effectively reduced this bias but slightly overcorrected, yielding densities lower than 3D measurements. We further evaluated the algorithm’s performance across varying sectioning intervals. Oocyte density estimates remained stable when section intervals ranged from 1 to 10. At longer intervals, estimates became increasingly inconsistent, reflecting the influence of heterogeneous follicle spatial distribution.

## Discussion

The number of females undergoing OTC is steadily increasing. According to a recent review of five leading European centers offering OTC, approximately 7,800 women had undergone the procedure by the year 2020 [33]. This trend is expected to continue as the number of centers providing the service expands and access becomes more widespread. The number and quality of primordial follicles in cryopreserved ovarian tissue are critical determinants of successful fertility restoration following transplantation. For samples intended for long-term storage, it is also essential to obtain robust estimations of tissue quality, including the average volume and morphology of cortical fragments. However, comprehensive screening of all archived samples using conventional morphometric methods is labor-intensive and impractical, and does not provide a representative overview of the stored material. In the present study, we demonstrate that micro-CT, combined with machine learning-based segmentation and morphometric image analysis, offers a high-throughput and automated method for quantifying oocyte density within the entire cortical sample. We successfully generated three-dimensional reconstructions of scanned ovarian tissue, enabling precise quantification and spatial localization of follicles in both adult and pediatric samples. In addition, the approach allowed for accurate volumetric measurement of individual tissue fragments and assessment of their cross-sectional morphology. Micro-CT imaging represents a promising alternative to conventional histology, with significant potential for both clinical application and research in the context of fertility preservation.

### Detection of oocytes and calculation of oocyte density

Previous studies have demonstrated the potential of micro-CT in ovarian tissue imaging [21–24], even detecting some early stage follicles in human ovarian cortical samples [24]. Additionally, protocols have been optimized by testing different staining protocols in bovine ovarian samples in order to efficiently preserve the morphological ovarian structure in the scanning [22]. By combining micro-CT imaging with an artificial intelligence (AI) algorithm we successfully generated three-dimensional reconstructions of the scanned ovarian tissues and oocytes, providing exact count and precise localization of preantral follicles. This method proved effective for both adult and pediatric ovarian tissue, capturing structural differences such as variations in follicle distribution and cortical thickness between age groups. Furthermore, the orientation of histological sections has a critical impact on the accuracy of follicle quantification. In cases where the surface epithelium is not included in the section, the cortical area may be misidentified, compromising the validity of the analysis [8]. In contrast, three-dimensional micro-CT imaging enabled complete visualization of the entire tissue volume, allowing for consistent, orientation-independent assessment of oocyte density and spatial distribution. Future research could expand the application of machine learning segmentation to detect additional structures beyond oocytes, such as blood vessels, within ovarian cortical tissue. The vascular architecture of the ovarian cortex is challenging to analyze using conventional 2D histology but 3D imaging combined with advanced segmentation could enhance the analysis of tissue quality and improve the assessment of cryopreserved ovarian samples.

### Distance of the oocytes from the ovarian surface epithelium

The primordial follicle pool in adults is located within the ovarian cortex [34], and current OTC protocols recommend preserving 1–2 mm thick cortical strips to retain this population [35]. However, it has remained unclear whether primordial follicles in prepubertal ovaries are similarly confined or located deeper within the stroma [36]. Present findings from high-resolution micro-CT imaging indicate that, in pediatric ovarian tissue, oocytes are located significantly closer to the ovarian surface compared to adult samples. This results in a thinner functional cortical layer in children, often less than 1 mm in thickness. These observations suggest that cortical strips of 1 mm thickness are sufficient to preserve the primordial follicle pool in both pediatric and adult ovarian tissue. The thinner functional cortical layer observed in children also impacts the definition of the cortical area used for follicle density calculations. Applying this standardized 1 mm cortical area to pediatric samples in mean follicle density (MFD) calculations may inadvertently include medullary regions, potentially leading to an underestimation of cortical follicle density compared to adult samples. These findings highlight the importance of considering age-specific cortical thickness in MFD assessments and suggest that the anatomical definition of the ovarian cortex in prepubertal individuals may need to be refined.

### Oocyte clustering

Oocyte clustering has been observed in both murine [37] and human ovaries, with the extent of primordial follicle clustering reported to increase with age [12]. Clustering has also been observed in prepubertal mice [38] and peripubertal human ovaries [11], suggesting that it originates during early developmental stages. During embryonic development, primordial germ cells undergo mitotic divisions, forming germ cell cysts composed of interconnected cells. These cysts subsequently break down to form individual primordial follicles, establishing the ovarian reserve [39, 40]. Clustering may also arise secondarily as a result of significant reductions in follicle number during childhood. Notably, a high proportion of morphologically abnormal follicles—characterized by indistinct germinal vesicle membranes and absent nucleoli—have been reported in prepubertal ovarian tissue. These abnormal follicles are significantly reduced prior to puberty and are generally absent in adult ovaries [41]. 3D visualization combined with mathematical modeling in the present study provided a novel and robust approach for detecting oocyte clustering—an analysis not achievable with the same accuracy using conventional two-dimensional histological techniques. Clustering was detected even in pediatric ovarian samples, although it was less pronounced than in adult tissue. This, combined with the prominent clustering of primordial follicles in adult ovaries, suggests that some follicle clusters formed in childhood may persist into adulthood. Clustering has been proposed to play a key role in regulating follicle activation through local inhibitory signaling. Resting follicles in close proximity are thought to produce paracrine signals that suppress activation, thereby maintaining the quiescent follicle pool. As overall oocyte density declines with age, the surrounding inhibitory environment weakens, making isolated follicles without neighboring dormant follicles more likely to initiate growth [38]. This accelerated follicle activation has been reported in premenopausal women [42].

### Simulated histology

In the present study, a histology based previously validated algorithm for adjusting follicle density calculations across varying sectioning intervals was applied to virtual sections generated from micro-computed tomography (micro-CT) datasets. This approach enabled a direct comparison between micro-CT–based three-dimensional quantification and virtual histological sectioning of identical tissue specimens. The findings indicate that oocyte densities derived from micro-CT 3D analysis closely correspond to those derived from virtual sections when every 10th section was analyzed across the entire tissue sample. However, such extensive histological sectioning is not practical in clinical settings, where oocyte counts are typically estimated from only the first five serial sections taken at consistent intervals. In pediatric ovarian tissue, the relatively uniform and high oocyte density across the cortical region appeared to allow accurate estimation from a limited number of virtual histological sections, yielding results comparable to those obtained from micro-CT analysis. In contrast, adult ovarian tissue exhibited reduced oocyte density and increased spatial heterogeneity, with marked follicle clustering. This variability significantly affected the accuracy of density estimates from limited virtual sections. Given the substantial heterogeneity in follicle distribution—even among tissue fragments obtained from the same individual [11]—micro-CT offers a more comprehensive and reliable assessment of total follicle number compared to standard histological methods. In addition, micro-CT–based analysis and virtual section evaluation further demonstrated that the follicle density estimation algorithm effectively compensates for the overestimation of oocyte counts by reducing the likelihood of duplicate counts. However, corrected oocyte density values consistently fell below those obtained from 3D micro-CT quantification. This discrepancy is likely due to an overcorrection within the algorithm, which assumes a uniform oocyte diameter based on the largest 10% of measured oocytes. The underestimation of oocyte density suggests systematic overmeasurement of oocyte diameters in the samples. Therefore, the optimal oocyte diameter used in the algorithm should be re-evaluated.

### Limitations

Scanning quality varied across samples. Four pediatric and one adult ovarian tissue sample were excluded due to poor image quality resulting from suboptimal staining or the presence of noise and artefacts in the micro-CT scans. This variability likely reflects a combination of technical and biological factors, including differences in sample preparation, staining efficiency, and intrinsic tissue properties. Notably, adult ovarian samples tended to exhibit greater stiffness than pediatric ones, which may have contributed to differences in imaging outcomes. For example, prolonged testosterone exposure in transgender patients has been associated with increased cortical stiffness in the ovary [43]. In addition, the extracellular matrix (ECM) composition differs between prepubertal and reproductive-aged ovaries [44], potentially influencing tissue texture and X-ray penetration during micro-CT imaging, and thereby affecting overall scan quality. Inconsistencies in scanning quality led to segmentation challenges, particularly in pediatric samples, where oocytes were detected and localized correctly but part of the oocytes were not fully segmented. As a result, reliable measurements of diameter, area and volume could not be obtained from full oocyte reserve. These limitations underscore the need to optimize scanning parameters tailored to the softer and more delicate nature of pediatric ovarian tissue. Further optimization could be achieved by integrating advanced 3D imaging modalities validated for murine *in toto* ovarian analysis, such as optical tissue clearing with light-sheet microscopy [45]. Correlating micro-CT data with quantitative measurements from immunolabeled oocytes would enable validation and refinement of the method’s accuracy.

Other limitations of the study included the limited sample size, primarily due to the restricted availability of ovarian tissue, particularly from pediatric patients. This also reduced the volume of data available for training the machine learning model, necessitating manual segmentation of selected sections for each sample. To address this, standard data augmentation techniques—such as image rotation, mirroring, and noise addition—were applied to expand the training and validation sets. While the imaging resolution was sufficient to detect primordial oocytes, it did not support accurate measurement of oocyte size or viability, thereby limiting the approach to quantitative analysis. However, morphological integrity alone does not reliably indicate reproductive potential, as recent studies have identified two viable subtypes of primordial follicles in the human ovary, only one of which exhibits gene expression profiles consistent with functional oocytes and granulosa cells [46]. For broader implementation in clinical practice, micro-CT-based analysis faces several limitations, primarily due to the high cost of scanners and the limited availability of personnel trained to operate the equipment and perform AI-based segmentation. It is unlikely to be feasible or cost-effective for each hospital to maintain an independent analysis facility. Therefore, we propose the establishment of centralized core units with the necessary infrastructure and expertise to perform standardized scanning and segmentation on samples collected from multiple institutions across broader geographic regions.

## Conclusions

Micro-CT combined with machine learning analysis shows strong potential as a reliable and efficient method for quantifying oocyte density in clinical fertility preservation. This approach could be integrated into standard protocols for ovarian tissue cryopreservation, with imaging data archived for potential future use. Moreover, it is compatiple with both fresh tissue and archived paraffin-embedded samples, broadening its applicability in both clinical and research settings. At its current stage of development, the method can detect oocytes from primordial follicles in both pediatric and adult ovarian samples. In adult ovarian tissue, the heterogeneous oocyte distribution enhances the accuracy of full-volume analysis compared to conventional histology. Given the higher follicle density and structural complexity in pediatric samples, the method is currently more applicable to adult tissue. Moreover, it enables quantification of spatial parameters—such as oocyte distance from the surface epithelium and local follicle clustering—offering valuable insights into cortical follicle organization. With continued methodological optimization, including expansion of sample size, optimization of tissue fixation and imaging protocols, and advancement of AI-based analytical models, the accuracy of the method can be improved, facilitating reliable quantitative assessment of oocyte size, morphology, and viability.

## Supplementary Information

Additional file 1.Additional Figure 1. Machine learning–based segmentation of oocytes in micro-CT virtual sections. Representative images from a pediatric sample (C1, age 2 years) and an adult sample (A6, age 39 years) are presented. The first column shows the original micro-CT acquired virtual section. The second column displays the manually annotated training dataset used as the ground truth for model training, with oocytes labeled in blue. The third column illustrates the corresponding output of the trained machine learning model, in which segmented oocytes are visualized in yellow.

Additional file 2. Additional Video 1. Three-dimensional visualization of a representative pediatric ovarian sample (C1).The sequence begins with a full rotation of intact tissue volume to illustrate overall morphology. Virtual sectioning is then applied, revealing oocytes as hypodense spots. These structures are subsequently segmented and highlighted in red. The surface epithelium is visualized in blue to delineate cortical boundaries. The sequence concludes with a magnified view of the segmented oocytes, followed by a return to the full-volume perspective.

Additional file 3. Additional Figure 2. Oocyte density assessment in adult and pediatric ovarian tissue using micro-CT and virtual sections. Representative data from an adult sample (A1) and a pediatric sample (C2) are shown. Oocyte densities were assessed using three approaches: full-volume 3D micro-CT analysis (red line), virtual histological sectioning at every tenth section throughout the entire tissue (green line), and from a limited sampling approach using only five virtual sections at 10-section intervals (black line). Density estimates, especially in the adult sample, varied depending on the starting point of the five-section series, reflecting spatial heterogeneity.

Additional file 4. Additional Figure 3. Influence of sectioning interval on histological estimation of oocyte density. Representative images from one adult sample (A1) and one child sample (C2) illustrate oocyte density derived from 3D micro-CT analysis (red line) compared with virtual section-based oocyte density estimates obtained with (black line) and without (green line) application of the correction algorithm (11). Density estimates without correction are substantially overestimated due to repeated counting of individual oocytes. Application of the correction algorithm reduces this artifact, yielding values that closely approximate the true 3D-derived oocyte density. 

Additional file 5. Additional Table 1. Comparison of segmentation performance metrics (DICE, Precision, Recall) between U-net model output and Avizo post-processing. DICE scores were generally higher following Avizo post-processing, with most samples exceeding the acceptable threshold of 0.7. Samples C1 and A3 showed comparable values between methods. Precision values consistently improved with post-processing. In contrast, Recall values tended to decrease due to a deliberate pixel-level shrinkage applied during post-processing to prevent 3D oocyte model merging. This shrinkage, typically one pixel layer, minimized redundancy and preserved spatial distribution. Sample A2 was excluded from the table due to the absence of detectable oocytes. Despite the trade-off in size accuracy, Avizo post-processing was retained in the pipeline to prioritize reliable oocyte count and localization.

## Data Availability

The raw and annodated datasets of this study are not publicly available due to data sensitivity and and patient confidentiality constraints but can be accessed from the corresponding author upon reasonable request. All data are securely maintained in controlled-access repositories at Karolinska Institutet and Helsinki University Hospital. The Python script used for model training and evaluation is likewise available from the corresponding author upon reasonable request.
