# Supplementary figures and images for: Micro-CT and machine learning: a high-throughput alternative to histology for follicle reserve assessment in cryopreserved ovarian tissue
Source: J Ovarian Res. 2025 Dec 23;19:15. doi: 10.1186/s13048-025-01897-8 (PMC12805687; doi:10.1186/s13048-025-01897-8)

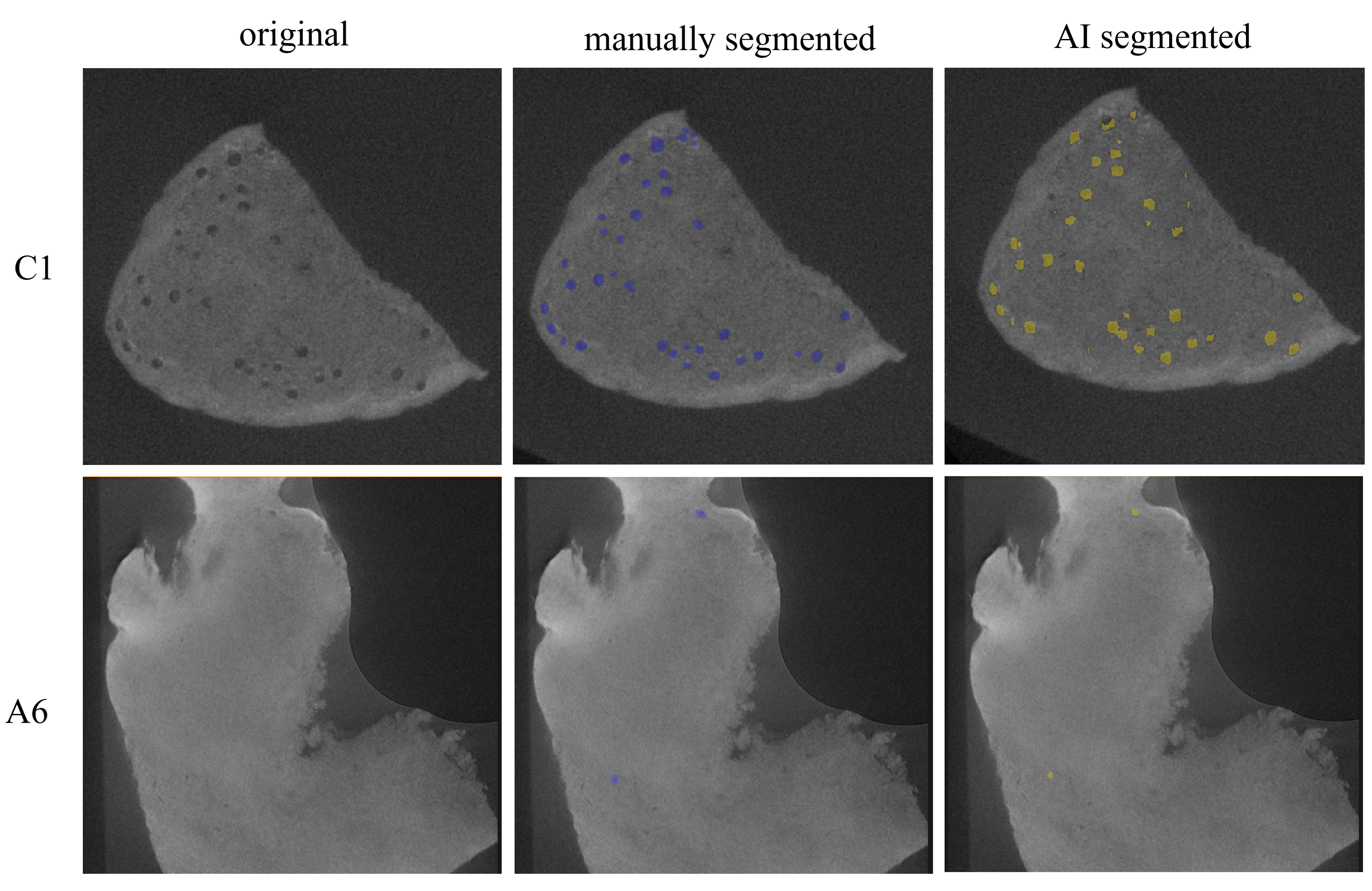

Supplement: Supplementary file 1 — Additional file 1.Additional Figure 1. Machine learning–based segmentation of oocytes in micro-CT virtual sections. Representative images from a pediatric sample (C1, age 2 years) and an adult sample (A6, age 39 years) are presented. The first column shows the original micro-CT acquired virtual section. The second column displays the manually annotated training dataset used as the ground truth for model training, with oocytes labeled in blue. The third column illustrates the corresponding output of the trained machine learning model, in which segmented oocytes are visualized in yellow. [file 13048_2025_1897_MOESM1_ESM.png]

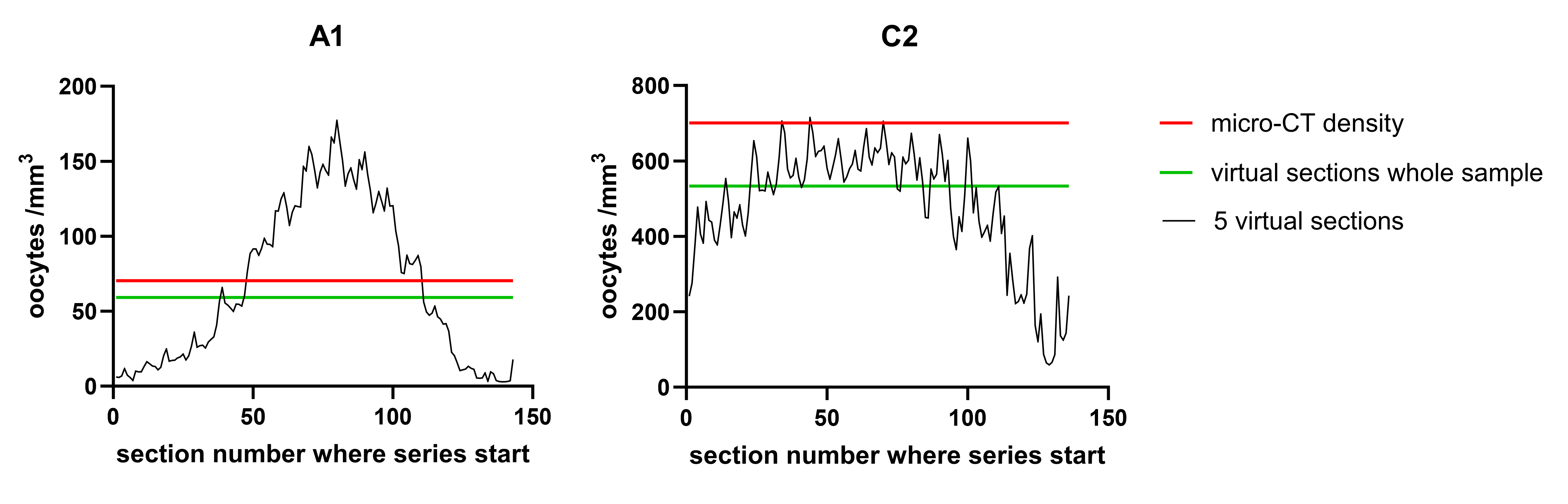

Supplement: Supplementary file 3 — Additional file 3. Additional Figure 2. Oocyte density assessment in adult and pediatric ovarian tissue using micro-CT and virtual sections. Representative data from an adult sample (A1) and a pediatric sample (C2) are shown. Oocyte densities were assessed using three approaches: full-volume 3D micro-CT analysis (red line), virtual histological sectioning at every tenth section throughout the entire tissue (green line), and from a limited sampling approach using only five virtual sections at 10-section intervals (black line). Density estimates, especially in the adult sample, varied depending on the starting point of the five-section series, reflecting spatial heterogeneity. [file 13048_2025_1897_MOESM3_ESM.tif]

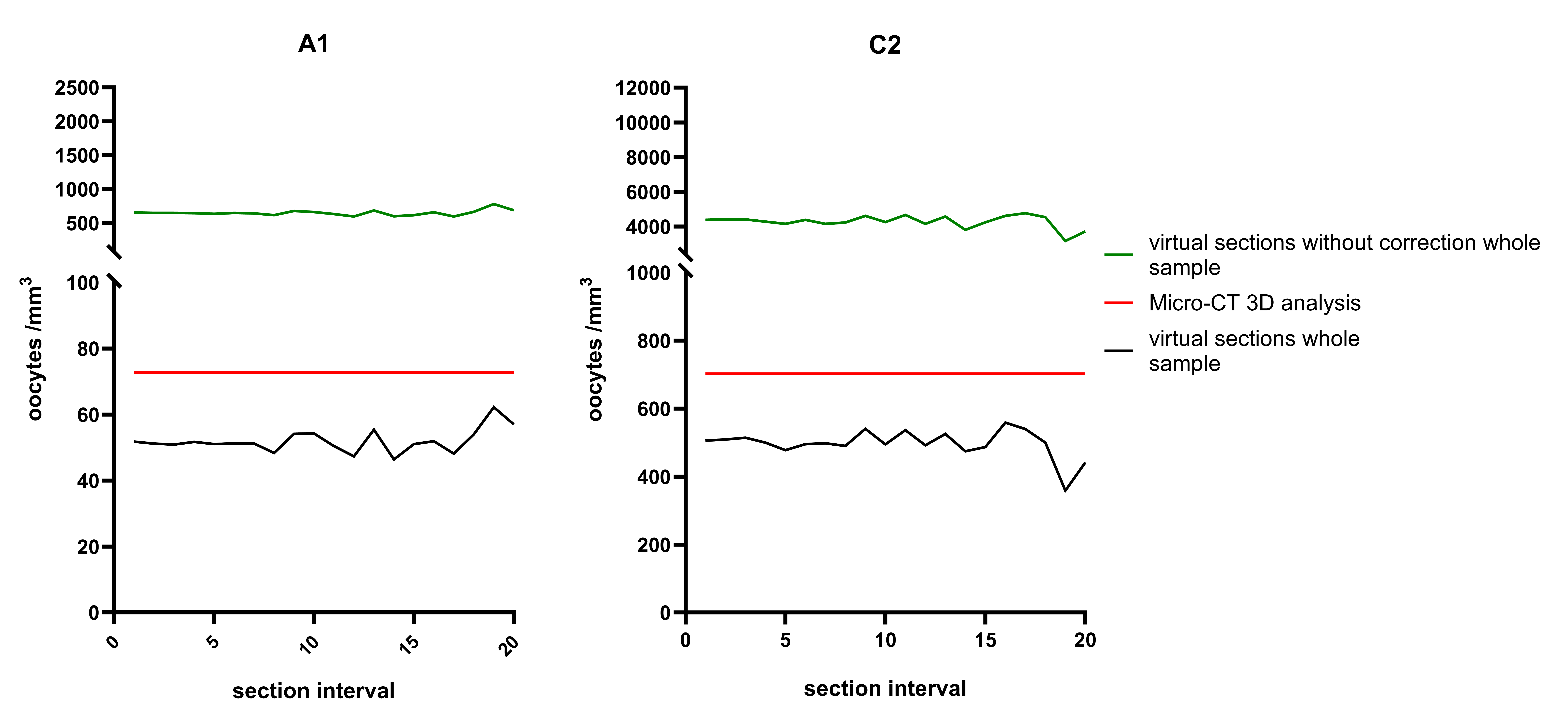

Supplement: Supplementary file 4 — Additional file 4. Additional Figure 3. Influence of sectioning interval on histological estimation of oocyte density. Representative images from one adult sample (A1) and one child sample (C2) illustrate oocyte density derived from 3D micro-CT analysis (red line) compared with virtual section-based oocyte density estimates obtained with (black line) and without (green line) application of the correction algorithm (11). Density estimates without correction are substantially overestimated due to repeated counting of individual oocytes. Application of the correction algorithm reduces this artifact, yielding values that closely approximate the true 3D-derived oocyte density. [file 13048_2025_1897_MOESM4_ESM.tif]
